# Predictive approaches to guide the expression of recombinant vaccine targets in *Escherichia coli*: a case study presentation utilising Absynth Biologics Ltd. proprietary *Clostridium difficile* vaccine antigens

**DOI:** 10.1007/s00253-021-11405-9

**Published:** 2021-06-28

**Authors:** Hirra Hussain, Edward A McKenzie, Andrew M Robinson, Neill A Gingles, Fiona Marston, Jim Warwicker, Alan J Dickson

**Affiliations:** 1 Manchester Institute of Biotechnology, University of Manchester, M1 7DN, Manchester, UK; 2 Absynth Biologics Ltd., BioHub, Alderley Park, Cheshire, SK10 4TG UK; 3 Present Address: Evotec Limited, Biohub, Alderley Park, Cheshire, England SK10 4TG UK; 4 Present Address: metaLinear Limited, Biohub, Alderley Park, Cheshire, England SK10 4TG UK; 5 Present Address: Liverpool School of Tropical Medicine, L3 5QA, Liverpool, UK

**Keywords:** Absynth Biologics Ltd., Recombinant protein production, Antigens, Bacterial expression, ‘Difficult-to-express’, Sequences and structural analysis, Predictive tools

## Abstract

Bacterial expression systems remain a widely used host for recombinant protein production. However, overexpression of recombinant target proteins in bacterial systems such as *Escherichia coli* can result in poor solubility and the formation of insoluble aggregates. As a consequence, numerous strategies or alternative engineering approaches have been employed to increase recombinant protein production. In this case study, we present the strategies used to increase the recombinant production and solubility of ‘difficult-to-express’ bacterial antigens, termed Ant2 and Ant3, from Absynth Biologics Ltd.’s *Clostridium difficile* vaccine programme. Single recombinant antigens (Ant2 and Ant3) and fusion proteins (Ant2-3 and Ant3-2) formed insoluble aggregates (inclusion bodies) when overexpressed in bacterial cells. Further, proteolytic cleavage of Ant2-3 was observed. Optimisation of culture conditions and changes to the construct design to include N-terminal solubility tags did not improve antigen solubility. However, screening of different buffer/additives showed that the addition of 1–15 mM dithiothreitol alone decreased the formation of insoluble aggregates and improved the stability of both Ant2 and Ant3. Structural models were generated for Ant2 and Ant3, and solubility-based prediction tools were employed to determine the role of hydrophobicity and charge on protein production. The results showed that a large non-polar region (containing hydrophobic amino acids) was detected on the surface of Ant2 structures, whereas positively charged regions (containing lysine and arginine amino acids) were observed for Ant3, both of which were associated with poor protein solubility. We present a guide of strategies and predictive approaches that aim to guide the construct design, prior to expression studies, to define and engineer sequences/structures that could lead to increased expression and stability of single and potentially multi-domain (or fusion) antigens in bacterial expression systems.

**Supplementary Information:**

The online version contains supplementary material available at 10.1007/s00253-021-11405-9.

## Key points

- *Model ‘difficult-to-express’ vaccine antigens were expressed in E. coli and characterised.*
- *Unfavourable sequence features potentially result in aggregation and/or improper processing.*
- *A workflow is described to guide expression of ‘difficult-to-express’ antigens in bacterial cells.*

## Introduction

Bacterial expression systems remain a widely used host for recombinant protein production. *Escherichia coli* (*E. coli*) remain the preferred used bacterial expression system, due to the low cost, rapid growth and high-cell densities achieved (Walsh 2018). However, disadvantages of using this system include the inability to perform eukaryotic post-translational modifications, e.g. glycosylation and di-sulphide bond formation for correct protein folding and protein maturation. Further, overexpression of recombinant target proteins in *E. coli* can result in poor solubility and in the formation of inclusion bodies (Demain and Vaishnav 2009; Kamionka 2011).

Inclusion bodies are enriched with insoluble denatured protein aggregates, which subsequently require additional extraction and re-folding processes to isolate proteins of interest. Proteins that localise to inclusion bodies usually have low or no biological activity. It has been reported that 15–25% of the total amount of protein is recovered from inclusion bodies, due to a loss of secondary structure and protein aggregation during solubilisation and re-folding processes (Kamionka 2011; Kaur and Kumar 2017). However, an advantage of protein purification from inclusion bodies includes the ease in isolating the inclusion body fraction via high-speed centrifugation from bacterial lysates (Georgiou and Valax 1999; Taylor et al. 1986). In addition, inclusion bodies can have a protective effect and prevent proteolytic degradation resulting in homogeneity of protein species within this fraction (Kaur and Kumar 2017; Oberg et al. 1994; Przybycien et al. 1994).

Many strategies or engineering approaches have been employed to increase recombinant protein production and overcome limitations in the production of ‘difficult’ or toxic target proteins in *E. coli* from the construct design through to the purification and formulation of target proteins (Gupta and Shukla 2016; Gupta and Shukla 2017; Kaur and Kumar 2017). Efficient recombinant protein production in *E. coli* relies on a combination of the correct DNA construct, host cell strain and downstream protein purification processes. Within each of these processes, there are multiple factors that can impact recombinant protein production. Strategies for optimal expression of targets rely on careful optimisation of these variables/factors and often are specific to each recombinant target. Examples of approaches used to improve protein production of ‘difficult’ recombinant targets include engineering an optimal combination of DNA elements in the expression vector. Also as part of the construct design, protease-cleavable solubility and/or detection tags have been used to aid protein expression and purification leading to increased amounts of protein recovered from bacterial cultures (Gupta and Shukla 2016; Jacquet et al. 1999; Martinez et al. 1995; Sorensen et al. 2003; Zheng et al. 2003).

Different *E. coli* host cell strains have been engineered and tailored to the requirements of the recombinant targets such as those capable of performing specific post-translational modifications (Gupta and Shukla 2016; Kaur and Kumar 2017; Schlegel et al. 2017). Another approach has been the co-expression of chaperones. Different groups of chaperones exist in *E. coli* to aid protein folding and prevent aggregation and/or degradation, and co-expression of these factors have shown to increase protein yields and solubility of certain recombinant targets (De Marco et al. 2007; Gupta and Shukla 2016; Nishihara et al. 1998). Great efforts have been focused on optimisation of the culture conditions such as the growth medium, inducer concentrations (Hemmerich et al. 2017; Ramirez et al. 1994), induction temperature (Barazesh et al. 2019; Schein and Noteborn 1988) and the isolation, re-solubilisation and purification of proteins from inclusion bodies (Kaur and Kumar 2017; Oberg et al. 1994; Przybycien et al. 1994). Other strategies employed include the targeting of proteins to different cellular compartments, for example, the periplasmic space where an oxidising environment and lower protease concentration has been shown to increase protein production and stability (Malik 2016). Alongside the optimisation of bacterial systems, computational approaches have allowed the prediction of sequence and/or structural features that may impact efficient production and solubility (Chennamsetty et al. 2009; Fernandez-Escamilla et al. 2004; Hebditch et al. 2017; Hebditch and Warwicker 2019; Rodrigues et al. 2021; Sormanni et al. 2015; Trainor et al. 2017). Such tools have allowed the re-design of recombinant targets with increased expression and/or solubility (Courtois et al. 2015; Perchiacca et al. 2011; Rodrigues et al. 2021; Sahin et al. 2011; Sormanni et al. 2015). Together, the optimisation of recombinant protein production tends to be tailored to each specific recombinant target, and therefore more generic predictive tools and/or guides to aid efficient recombinant protein production would be beneficial.

Absynth Biologics Ltd. is focused on the discovery and development of novel vaccines that target a range of infectious diseases to address the challenge of antimicrobial resistance. Absynth Biologics Ltd. has a portfolio of proprietary antigens that have been identified in *Clostridium difficile* (*C. difficile*) as potential vaccinogens. Two leading antigens, Ant2 and Ant3, are small protein domains taken from essential proteins that localise to the bacterial outer cell membrane. Ant2 was taken from a protein ortholog of tRNA N6-adenosine threonylcarbamoyltransferase (YdiE/TsaD) (Deutsch et al. 2012; Lauhon 2012; Luthra et al. 2019; Missoury et al. 2018; Zhang et al. 2015). TsaD is a predicted membrane protein that has been assigned multiple functions that include RNA translational fidelity and regulation of membrane transport and may act as a protease (Zhang et al. 2015; Zheng et al. 2005). Whereas, Ant3 was taken from a bacterial cell division protein (DivIB/FtsQ), that has been shown to bind to the cell wall and is involved in bacterial morphogenesis and cell division (Bottomley et al. 2014; Choi et al. 2018; De Boer 2010; Kureisaite-Ciziene et al. 2018; Lutkenhaus and Addinall 1997; van den Ent et al. 2008).

Earlier expression and purification studies of Ant2 and Ant3 antigens (from *C. difficile*) by Absynth Biologics Ltd. showed that overexpression of both proteins produced insoluble protein (inclusion bodies). Proteins isolated from the inclusion bodies had an increased tendency to oligomerise, and protein precipitation was observed during the purification steps (*unpublished work*). This case study presents the strategies used to increase the recombinant production and solubility of these ‘difficult-to-express’ bacterial proteins, Ant2 and Ant3, from Absynth Biologic Ltd’s *C. difficile* portfolio. We also present a workflow or ‘fast-track’ guide with the use of predictive approaches that aim to guide the construct design and increased expression of single and potentially multi-domain (or fusion) antigens in bacterial expression systems.

## Material and methods

### Materials

All reagents used were of the highest grade and purchased from Sigma-Aldrich unless stated otherwise.

### DNA constructs

Two protein sequences from Absynth Biologics Ltd.’s portfolio of antigens from *C. difficile*, Ant2 (YdiE/ TsaD) and Ant3 (DivIB/FtsQ), were cloned into the pET21d(+) expression vector (Novagen) using *Nco*I (5’) and *Xho*I (3’) restriction sites. All sequences used were codon-optimised for bacterial expression. The GenBank accession numbers for the nucleotide sequences used for Ant2 and Ant3 were CP028529.1 and CP011847.1, respectively. The GenPept accession numbers for the protein sequences used for Ant2 and Ant3 were WP_021389366 and WP_131025834.1, respectively. In addition, both protein sequences were joined in different orientations to form fusion proteins, fusion 1 (Ant2-Ant3, Ant2-3) and fusion 2 (Ant3-Ant2, Ant3-2) and cloned separately into the pET21d(+) vector. All constructs contained a C-terminal 6×His tag for detection and purification.

The coding sequence for fusion 2 (Ant3-2) was cloned into three different pET16 parental expression vectors (provided by EA McKenzie) with a cleavable N-terminal 6×His tag alone and in combination with a thioredoxin (Trx) or N utilisation substance protein A (NusA) solubility tag (termed pHis, pHisTrx and pHisNusA, respectively). Ant3-2 coding sequences were amplified by polymerase chain reaction (PCR, Table 1) and cloned into the three parental expression vectors using *Bam*HI (5’) and *Eco*RI (3’) restriction sites. *Bam*HI and *Eco*RI restriction sites were introduced into PCR product via the forward and reverse primer respectively (Table 1). Sequences for Ant2, Ant3 and fusion 1 (Ant2-3) were amplified by PCR using primers designed using the In-Fusion® primer design tool (Table 1) and cloned into pHisNusA using the In-Fusion® HD cloning method (Clontech, cat no. 638916) as per the manufacturers’ instructions.

**Table 1:** Summary of primers used for PCR amplification of antigen coding sequences.

Primer		Sequence (5′-3′)
Ant2	Forward	GTACTTCCAG**GGATCC**AATCACATCGAAGGCCATCTGT
	Reverse	CCGGATCTTA**GAATTC**TTATTATCCCTCCGGAGAGTATACCGC
Ant3	Forward	GTACTTCCAG**GGATCC**ATGGCAAATCATATAGAAGG
	Reverse	CCGGATCTTA**GAATTC**TTATTACTGATTTATCTTTAAATTTGG
Fusion 1 (Ant2-3)	Forward	GTACTTCCAG**GGATCC**ATGGCAGTAAAGAAAATAGA
	Reverse	CCGGATCTTA**GAATTC**TTATTATCCTTCTGGACTATACACTG
Fusion 2 (Ant3-2)	Forward	CAC**GGATCC**GTCAAAAAGATTGATGTGATTG
	Reverse	CAC**GAATTC**TTATTACTGATTAATTTTCAGGTTTG

This table summarises the forward and reverse primers for PCR amplification of each antigen coding sequence. Restriction sites introduced in the forward (*Bam*HI) and reverse (*Eco*RI) primer restriction sites (bold) and overhangs generated for In-Fusion® HD cloning (underlined) are highlighted

### Small-scale bacterial expression

DNA constructs for the single antigens (Ant2 and Ant3) and fusions (Ant2-3 and Ant3-2) were transformed into BL21-CodonPlus (DE3) *E. coli* cells. A single colony was used to inoculate 5 ml overnight culture (LB broth with 100 μg/ml ampicillin). The next day, bacterial cultures were seeded from the overnight culture (1:100 dilution) in a total volume of 100 ml LB broth containing ampicillin (100 μg/ml final concentration). The cells were grown at 37°C with shaking at 220 rpm. The optical density at 600 nm (OD_600_) for each culture was monitored until an OD_600_ of 0.5–0.7 was reached. The expression of the recombinant antigens was induced with isopropyl β-D-1-thiogalactopyranoside (IPTG, 0.2 mM final concentration), followed by incubation at 37°C, 30°C or 18°C for 20 h at 220 rpm. For growth at lower temperatures (30°C and 18°C), cultures were cooled to the appropriate temperature prior to induction. Cultures were harvested by centrifugation (4000 rpm, 4°C for 20 min). Cell pellets were re-suspended in 4 ml ice-cold lysis buffer (25 mM Tris-HCl pH 7.9, 0.3 M NaCl, 1% (v/v) Triton-X100, 0.2% (v/v) protease inhibitor cocktail) and sonicated on ice (7 cycles, 30 s on/off pulses at 35% amplitude, QSonica sonicator ultrasonic processor, Q125). After lysis, an aliquot (40 μl) of the bacterial lysate (total fraction) was isolated for SDS-PAGE and western blot analysis. The insoluble and soluble fractions were isolated by centrifugation (15,000 rpm, 4°C for 15 min). Aliquots (40 μl) of the soluble fraction (supernatant) and insoluble fraction (pellet) were isolated for SDS-PAGE and western blot analysis.

### Large-scale bacterial expression

DNA constructs were transformed into BL21-CodonPlus (DE3) *E. coli* cells. A single colony was used to inoculate a 5 ml starter culture (LB broth with 100 μg/ml ampicillin) and incubated at 37°C, 220 rpm for ~6 h. The overnight culture was inoculated with 50 μl of the starter culture in 50 ml selective LB broth (1:1000 dilution) and incubated overnight at 37°C with shaking at 220 rpm. The next day, bacterial cultures were seeded from the overnight culture (1:100 dilution) in a total volume of 1 l LB broth containing ampicillin (100 μg/ml final concentration). The cells were grown at 37°C with shaking at 220 rpm. At the correct OD_600_ (0.5–0.7), cultures were cooled (4°C for 30 min) and induced with IPTG (0.2 mM final concentration) for 20 h at 18°C, 220 rpm. Cultures were harvested by centrifugation (5000 rpm, 4°C for 10 min). The dry pellet weight was recorded, and cell pellets were re-suspended in 30 ml ice-cold lysis buffer and sonicated on ice (7 cycles, 30 s on/off pulses at 25% amplitude, Bandelin Sonoplus sonicator, HD3200). After lysis, an aliquot (40 μl) of the bacterial lysate (total fraction) was isolated for SDS-PAGE and western blot analysis. The insoluble and soluble fractions were isolated by centrifugation (17,000 rpm, 4°C for 30 min). Aliquots (40 μl) of the soluble fraction (supernatant) and insoluble fraction (pellet) were isolated for SDS-PAGE and western blot analysis.

### Protein re-folding

After high-speed centrifugation of the total fraction, the isolated inclusion body–enriched pellet (insoluble fraction) was used for protein re-folding purposes. The pellet was re-suspended in either 4 ml (small-scale) or 30 ml (large-scale) urea buffer (5 mM imidazole, 1 M NaCl, 40 mM Tris-HCL pH 7.9, 8 M urea and 0.14% (v/v) β-mercaptoethanol) and sonicated (5 cycles, 30 s on/off pulses at 25% amplitude). The suspension was centrifuged at 17,000 rpm, 18°C for 30 min to remove cell debris. The supernatant was isolated for purification.

### His-tag purification

For small-scale purification (100 ml bacterial culture) from the soluble fraction, 1 ml Ni-NTA agarose (50% suspension, Qiagen) was washed twice with 14 ml ice-cold phosphate buffered saline (PBS) buffer in a 15 ml falcon tube. For large-scale purifications (1 l bacterial culture), 5 ml Ni-NTA agarose was washed twice with 45 ml ice-cold PBS in a 50 ml falcon tube.

### Purification from the soluble fraction

All incubation steps were carried out at 4°C and spin steps completed at 4000 rpm, 4°C for 5 min (small-scale) or 10 min (large-scale).

For small-scale purification, the Ni-NTA resin was equilibrated with 2 ml ice-cold lysis buffer and then spun down and supernatant was discarded. Four millilitres suspension (soluble fraction) was added to the resin and incubated on a roller for 2 h at 4°C. After incubation, the suspension was spun down and the flow through collected. The resin was washed three times with increasing imidazole concentrations, first with 10 ml buffer 1 (25 mM Tris-HCl pH 7.9, 0.3 M NaCl, 5 mM imidazole, 0.2% (v/v) protease inhibitor cocktail), then 10 ml buffer 2 (25 mM Tris-HCl pH 7.9, 0.3 M NaCl, 10 mM imidazole) and a third time with 10 ml buffer 3 (25 mM Tris-HCl pH 7.9, 0.3 M NaCl, 15 mM imidazole) for 1 min each. After each wash, the suspension was spun down and supernatant (washes 1–3) isolated. Soluble proteins were eluted with elution buffer (25 mM Tris-HCl pH 7.9, 0.3 M NaCl, 250 mM imidazole) in 3 × 1 ml steps (E1-E3) at 4°C. All eluates were stored at 4°C.

For large-scale purification, the Ni-NTA resin was equilibrated with 10 ml ice-cold lysis buffer and then spun down and supernatant was discarded. Thirty millilitres suspension (soluble fraction) was added to the resin and incubated on a roller for 2 h at 4°C. After incubation, the suspension was spun down and the flow through collected. The resin was washed three times consecutively with 50 ml each of buffer 1, buffer 2 and buffer 3 for 20 min at 4°C. After each wash, the suspension was spun down and supernatant isolated. Soluble proteins were eluted with elution buffer in 9 × 1 ml steps (E1-E3) at 4°C.

At each stage, 100 μl aliquots of each fraction (flow through, washes and eluates) were collected for protein concentration determination, SDS-PAGE and western blot analysis.

### Purification from the insoluble fraction

For small-scale purifications (100 ml bacterial culture), all spin steps were carried out at 4000 rpm, 4°C or room temperature for 5 min. The resin was equilibrated with 2 ml urea buffer and then spun down. The supernatant was discarded. Four millilitres suspension (pellet/insoluble fraction) was added to the resin and incubated on a roller overnight at room temperature. The next day, the suspension was spun down and the flow through collected. The resin was washed with 10 ml buffer A (5 mM imidazole, 1 M sodium chloride, 20 mM Tris-HCL pH 7.9, 8 M urea) for 1 min at room temperature. The suspension was spun down and supernatant (wash 1) isolated. The resin was washed with 10 ml buffer B (5 mM imidazole, 1 M sodium chloride, 20 mM Tris-HCL pH 7.9 and 0.14% (v/v) β-mercaptoethanol) for 1 min at room temperature. The suspension was spun down and supernatant (wash 2) isolated. The resin was washed a third time with 10 ml buffer C (60 mM imidazole, 0.5 M sodium chloride, 20 mM Tris-HCL pH 7.9 and 0.14% (v/v) β-mercaptoethanol) for 1 min at 4°C. The suspension was spun down at 4°C and supernatant (wash 3) isolated. Re-folded proteins were eluted with buffer D (1 M, 0.5 M sodium chloride, 20 mM Tris-HCL pH 7.9 and 0.14% (v/v) β-mercaptoethanol) in 3 × 1 ml steps (E1-E3) at 4°C. Insoluble proteins bound to the resin (after re-folding) were eluted with a denaturing elution buffer, buffer E (5 mM imidazole, 1 M sodium chloride, 20 mM Tris-HCL pH 7.9, 8 M urea, EDTA) in 3 × 1 ml steps (DE1- DE3) at room temperature. All eluates were stored at 4°C.

For large-scale purifications (1 l bacterial culture), all spin steps were carried out at 4000 rpm, 4°C or room temperature for 10 min. Following the washes, the resin was equilibrated with 10 ml urea buffer and then spun down. The supernatant was discarded. Thirty millilitres ml suspension (pellet/insoluble fraction) was added to the resin and incubated on a roller overnight at room temperature. The next day, the suspension was spun down and the flow through collected. The resin was washed consecutively with 50 ml buffer A then buffer B for 20 min at room temperature on a roller. After each wash, the suspension was spun down and supernatant isolated. The resin was washed a third time with 50 ml buffer C for 20 min at 4°C on a roller. The suspension was spun down at 4°C and supernatant isolated. The resin was transferred to an empty single gravity flow chromatography column, and re-folded proteins were eluted with buffer D in 5 × 1 ml steps (E1-E5) at 4°C. Insoluble proteins (after-re-folding) were eluted with buffer E in 4 × 1 ml steps (DE1-DE4) at room temperature. All eluates were stored at 4°C.

At each stage, an aliquot (100 μl) of each fraction was collected for protein concentration determination and characterisation by SDS-PAGE and western blot.

### Determination of protein concentration

An estimate of the protein concentration was determined for purified samples using the Bio-Rad DC™ Protein Assay kit as per the manufacturers’ instructions.

### SDS-PAGE and western blotting

SDS-PAGE (12.5% (w/v)) gels were prepared and run using the Bio-Rad mini PROTEAN Tetra system. Protein samples were mixed in equal volume with 2× loading buffer (125 mM Tris-HCl, 4% (w/v) SDS, 20% (v/v) glycerol, 0.01% (v/v) bromophenol blue). Under reducing SDS-PAGE conditions, 2×-sample buffer was supplemented with 1.8% (v/v) β-mercaptoethanol. Samples were heated to 100°C for 5 min and cooled before loading. The following total volume of sample was loaded, 5 μl of the total induced (T), pellet/insoluble (P) and soluble (S) fraction and Bovine serum albumin (BSA) protein standard. For purified protein samples, 15 μl total volume was loaded. SDS-PAGE was performed in electrode buffer (25 mM Tris base, 190 mM glycine, 0.1% w/v SDS, pH 8.3). The marker and samples were electrophoresed at 60 V through the stacking gel and then 200 V until the dye front reached the bottom of the gel.

For western blot analysis, proteins separated by SDS-PAGE were transferred onto nitrocellulose membrane using the TE 22 wet transfer unit at 300 mA for 2 h. Blots were blocked for 1 h at room temperature in 5% (w/v) non-fat milk in phosphate buffered saline (PBS, 137 mM NaCl, 2.7 mM KCl, 10 mM Na_2_HPO_4_, 2 mM KH_2_PO_4_, pH 7.4) with 0.1% (v/v) Tween-20 (5% mPBS-T). A mouse monoclonal anti-6×His tag primary antibody (1:4000 dilution, Abcam, ab18184) and donkey anti-mouse secondary antibody (1:15,000, LI-COR Biosciences, 926-32212) were prepared in 5% mPBS-T and incubated with blots for 1 h at room temperature. Blots were imaged using the LI-COR Odyssey® Classic imager according to the manufacturer’s instructions. Analysis and quantification of bands was completed using the LI-COR Image Studio™ Lite software.

### Protein identification

Protein samples were separated by SDS-PAGE and submitted to the protein identification service (Manchester Institute of Biotechnology, University of Manchester). Protein samples were gel extracted, trypsin-digested and analysed by Dr. Martin Read using mass spectroscopy.

### Protein solubility screen

The solubility of purified protein samples was profiled in different formulations using the Optisol™ III protein soluble screening kit (Soluble Bioscience) as per the manufacturers’ instructions. Ant2 and Ant3 were expressed in the BL21-CodonPlus (DE3) *E. coli* strain (1 l total volume), and proteins were re-solubilised in urea buffer from the insoluble fraction and purified via the 6×His-tag. Proteins were eluted and the protein concentration was determined (Table SI). The top three concentrated samples were pooled for screening. A blank absorbance reading (280 nm) was taken of the plate with reagent alone. Fifteen microlitres of the pooled protein sample was mixed with 150 μl of reagent per well and incubated at 37°C for 24 h (stressed conditions). Following incubation the soluble protein was collected by centrifugation (3000 rpm, 30 min). The absorbance was measured after isolation of the soluble protein and the blank subtracted from these values. The data was subsequently analysed using the Protein Dashboard™ (supplied by Soluble Bioscience).

### Use of predictive tools for sequence- and structural-based analysis of antigens

Amino acid sequences for all antigens were analysed using JPred 4 (protein secondary structure prediction server) (Drozdetskiy et al. 2015) and ProteinSol (predictive protein solubility tool) (Hebditch et al. 2017; Hebditch and Warwicker 2019).

Three-dimensional structural models for Ant2 and Ant3 sequences were generated in SWISS-MODEL (Arnold et al. 2006; Bordoli et al. 2009). Models were based on sequence homology of both *C. difficile* Ant2 and Ant3 sequences from this study with published structures, in the protein data bank (PDB), of their corresponding *E. coli* homologs, YdiE (PDB ID: 4wq4) and FtsQ (PDB ID: 2vh1), respectively. Structural models for fusion proteins (Ant2-3 and Ant3-2) could not be generated. The structural models for Ant2 and Ant3 were analysed using the ProteinSol algorithm (in collaboration with Dr. J Warwicker, University of Manchester). The algorithm was used for a sequence-based solubility prediction and a structural-based prediction where surface mapping of model structures were analysed in terms of electrostatic potential (positive vs. negative charge) and hydrophobicity (polar vs. non-polar).

### Data analysis

All results are presented as the mean ± standard error of the mean (SEM) for at least three biological replicates. All graphs were plotted in GraphPad Prism® (Version 6.02).

## Results

### Characterisation of bacterial growth and recombinant protein production

Two antigens from Absynth Biologics Ltd.’s *C. difficile* portfolio, Ant2 and Ant3, were used as models to assess the effect of different strategies to improve production and solubility of these ‘difficult-to-express’ targets, together with predictive approaches to gain insight to whether these could be used to guide the design of antigens to aid increased recombinant production. As well as the single Ant2 and Ant3 antigens, fusion proteins were generated. The fusion proteins were designed to incorporate Ant2 and Ant3, in different orientations, Ant2-3 (fusion 1) and Ant3-2 (fusion 2), into a single polypeptide chain. As historical data from Absynth Biologics Ltd. showed Ant2 and Ant3 localised to the insoluble (inclusion body) fraction and recovered poorly (data not shown), we hypothesised that the fusions may act to improve production and solubility. In addition, the use of fusion protein would decrease the overall cost-of-goods by expressing both antigens simultaneously.

The codon-optimised DNA sequence of all antigens, Ant2, Ant3 and Ant2-3 (fusion 1) and Ant3-2 (fusion 2) (Figure S1), were synthesised in the bacterial expression vector, pET21d(+). A C-terminal His-tag was incorporated in all constructs for purification and detection purposes (Figure S1).

Preliminary work focused on the optimisation of a high-yielding bacterial expression protocol. A panel of *E. coli* strains (BL21 (DE3), BL21-CodonPlus (DE3), JM109 (DE3) and JM109-pGJKE8 (DE3)) was tested to evaluate their effect on the production of these antigens. Regardless of the strain used, all four antigens localised to the insoluble fraction, and this observation was not specific to the host cell strain (data not shown). For further studies, BL21-CodonPlus *E. coli* cells were selected as they were shown to express the largest amount of total protein of all the strains tested.

**Fig. 1:**
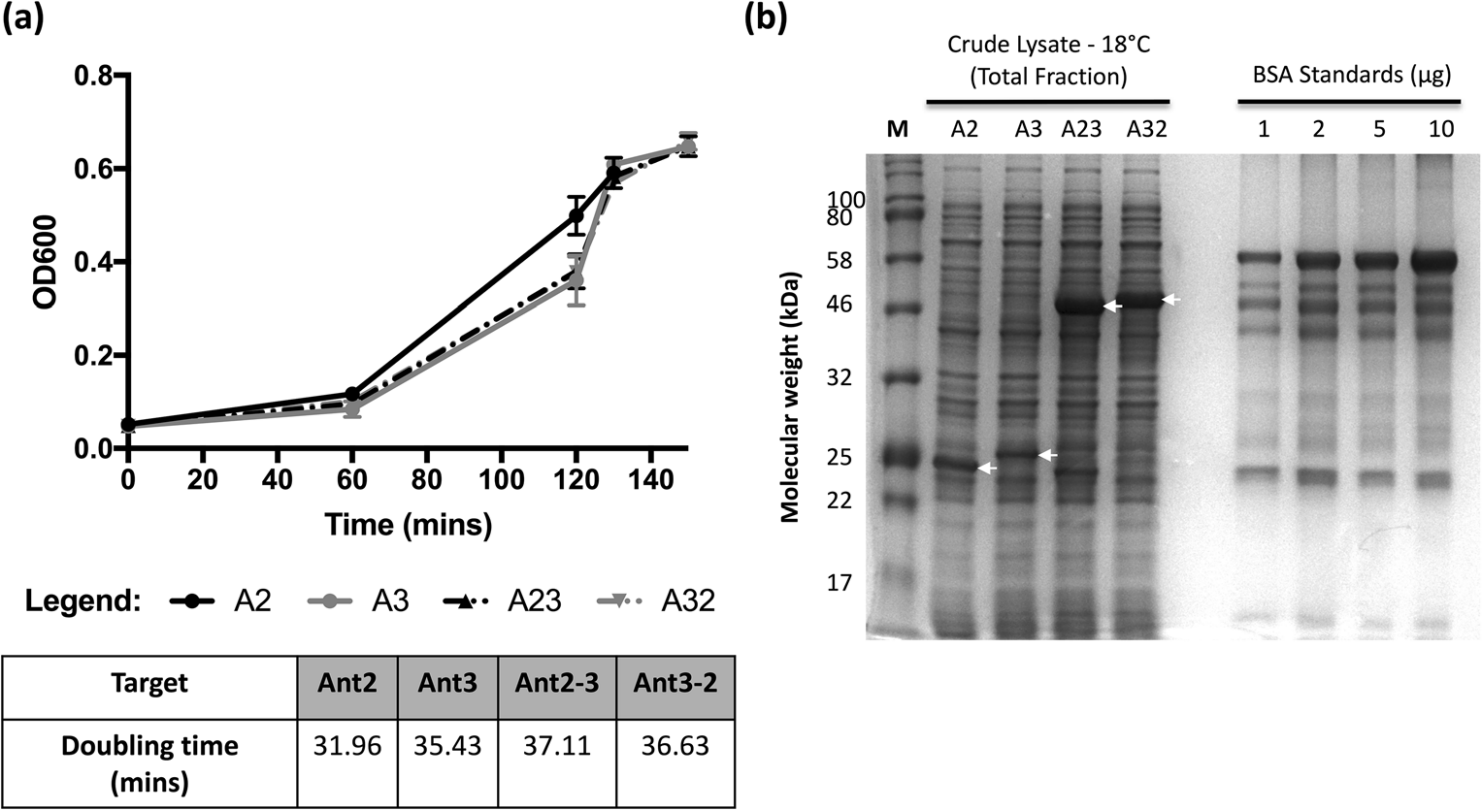
Analysis of growth and antigen production in bacterial cultures. DNA constructs for Ant2 (A2), Ant3 (A3), Ant2-3 (A23) and Ant3-2 (A32) were transformed into BL21-CodonPlus (DE3) *E. coli* cells. Bacterial cultures were seeded from overnight cultures (1:100 dilution) in a total volume of 100ml of LB media and the optical density monitored at 600 nm (OD600) prior to induction. This figure shows the OD600 plotted against the time (min) for bacterial cultures transformed with all four target antigens (**a**). The table summarises the estimated doubling time (min) of each culture analysed using the exponential growth equation in GraphPad Prism. Error bars shown are the mean value ± SEM of three biological replicates (n=3). Bacterial cultures for each antigen were harvested post-IPTG induction (20 h at 18°C). The cell pellet was lysed via sonication and a sample of the crude lysate (total fraction) was analysed by SDS-PAGE (**b**). Bovine serum albumin (BSA) was used as a protein standard. The band position for each antigen is indicated with an arrow. Data is representative of at least four biological replicates

The constructs for Ant2, Ant3 and Ant2-3 (fusion 1) and Ant3-2 (fusion 2) were transformed into the BL21-CodonPlus *E. coli* strain. Prior to induction, the bacterial growth was monitored at 37°C (Fig. 1a). No significant difference was observed between the growth/doubling times of all four antigens. Overexpression of the antigens was induced with IPTG for 20 h at three different temperatures 18°C, 37°C or 30°C. Post-induction, the bacterial crude lysates (total fraction, T) were analysed by SDS-PAGE. At 18°C, the single antigens, Ant2 (~25 kDa) and Ant3 (~26 kDa), were expressed in smaller amounts compared to the fusions, Ant2-3 and Ant3-2 (~51 kDa) (Fig. 1b). Parallel expression studies at the higher growth temperatures 37°C and 30°C showed an increase in the amount of the total protein but no effect on protein solubility (Figure S2).

### Assessment of protein solubility

**Fig. 2:**
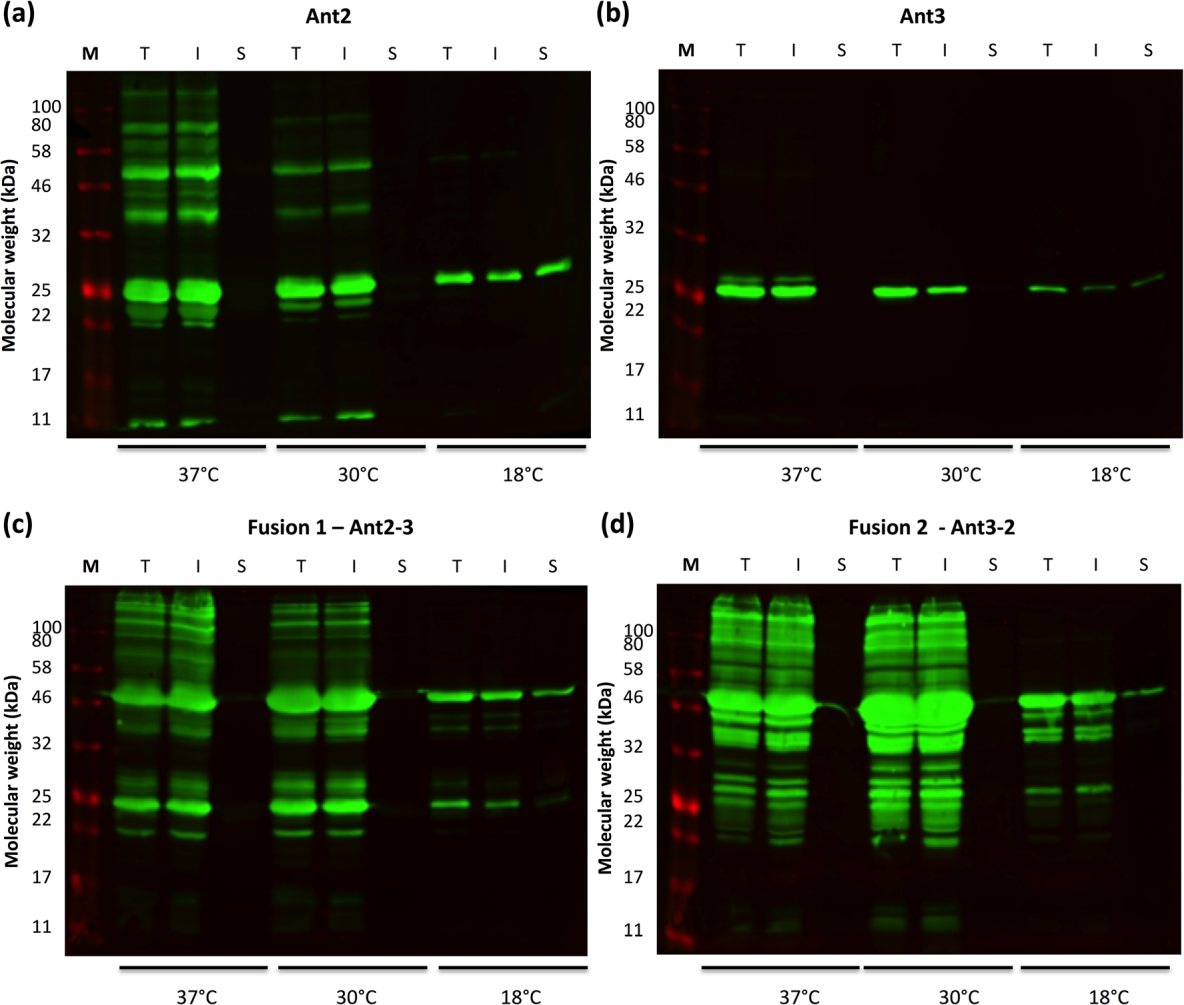
Assessment of protein solubility. Bacterial cultures grown at different temperatures (37°C, 30°C and 18°C) were harvested post-induction. The crude cell lysate (total fraction, T) was spun at high-speed (17,000 rpm, 4°C for 30 min) to isolate the insoluble fraction (I, pellet) and soluble fraction (S, supernatant). Each fraction were subsequently analysed by western blot to assess solubility. A 6×His-tag primary antibody was used for detection of the single antigens (Ant2 (**a**) and Ant3 (**b**)) and fusions (Ant2-3 (**c**) and Ant3-2 (**d**)). Data is representative of three biological replicates

To assess protein solubility, the total crude fraction (T) was centrifuged at high speed (17,000 rpm) to isolate the insoluble (I, pellet) and soluble (S, soluble) fraction. Aliquots of each fraction were analysed by western blotting, using a 6×His tag primary antibody was used for detection (Figure 2). Assessment of the protein solubility for all four antigens showed that although a greater amount of protein was detectable at the higher growth temperatures (37°C and 30°C), almost all the protein was detectable in the insoluble fraction. Further, a larger presence of high and low molecular weight species (predicted oligomers and degradation products) was observed at 37°C and 30°C. At 18°C, soluble protein was detectable for all antigens, and significantly less high and low molecular weight species were detected at the lower growth temperature (Fig. 2).

All four antigens showed a tendency to form oligomers. Presence of oligomers was detected by western blotting under non-reducing conditions (data not shown). In addition, lower molecular weight bands were also detected which may correspond to degradation products.

### Protein re-folding and His-tag purification

**Fig. 3:**
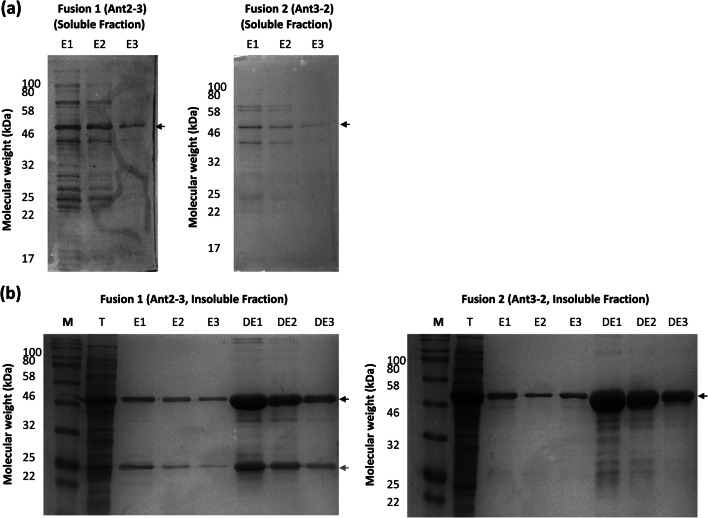
Purification of fusions from the soluble and insoluble fraction using immobilised metal affinity chromatography (IMAC). The fusion proteins were purified using the 6×His-tag via IMAC from the soluble (**a**) and insoluble (**b**) fraction. For the insoluble fraction, proteins were re-solubilised in urea buffer prior to 6×His-tag purification. Proteins were eluted with imidazole in 3 × 1 ml stages (E1-E3). For the insoluble fraction, after elution with imidazole (E1-E3), insoluble proteins (after the re-folding stage) were eluted in 3 × 1 ml stages with a denaturing elution buffer (DE1-DE3). The band position for each antigen is indicated with an arrow, and the predicted degradation product of fusion 1 is highlighted with a  grey arrow

To compare the yields and purity between the insoluble and soluble fraction, the antigens were purified from each fraction separately via the C-terminal 6×His-tag (Fig. 3 and Figure S3). As seen with the solubility data, a significant proportion of protein localised to the inclusion body–enriched insoluble fraction; therefore, proteins were first re-folded and subsequently purified.

A poor recovery of protein was observed from the soluble fraction for both fusions Ant2-3 and Ant3-2 (Fig. 3a). In addition, other unknown high and low molecular weight species were detected in the purified samples. These species could correspond to host cell proteins co-purifying with each antigen or non-specifically binding to the Ni-NTA agarose. In contrast, a larger amount of protein was recovered after re-solubilisation (Fig. 3b), though a significant proportion remained insoluble after re-solubilisation (fractions DE1-DE3) for both Ant2-3 and Ant3-2.

The same pattern was observed for the purification of single antigens. A larger amount was recovered from the insoluble fraction compared to the soluble fraction (Figure S3). However, for both Ant2 and Ant3 after protein re-folding from the insoluble fraction, almost all the protein was solubilised (Figure S3b). The data for the single antigens differed to the fusion data as a significant proportion of both fusion proteins remain insoluble after re-folding (Fig. 3b).

Post-6×His-tag purification of Ant2-3, a prominent band was detected at half the molecular weight (~25 kDa) of the full fusion protein in both soluble and insoluble fractions and which was not present in the Ant3-2 samples (Fig. 3). Protein identification of the low molecular weight band by mass spectroscopy showed that the band corresponded to a mixture of Ant2 and Ant3 antigens. The trypsin-digested protein fragments detected by mass spectroscopy mapped to either side of the boundary of Ant2 and Ant3 in the full fusion protein suggesting that improper processing of the fusion 2 was occurring (discussed further in the next section).

### Protein stability

**Fig. 4:**
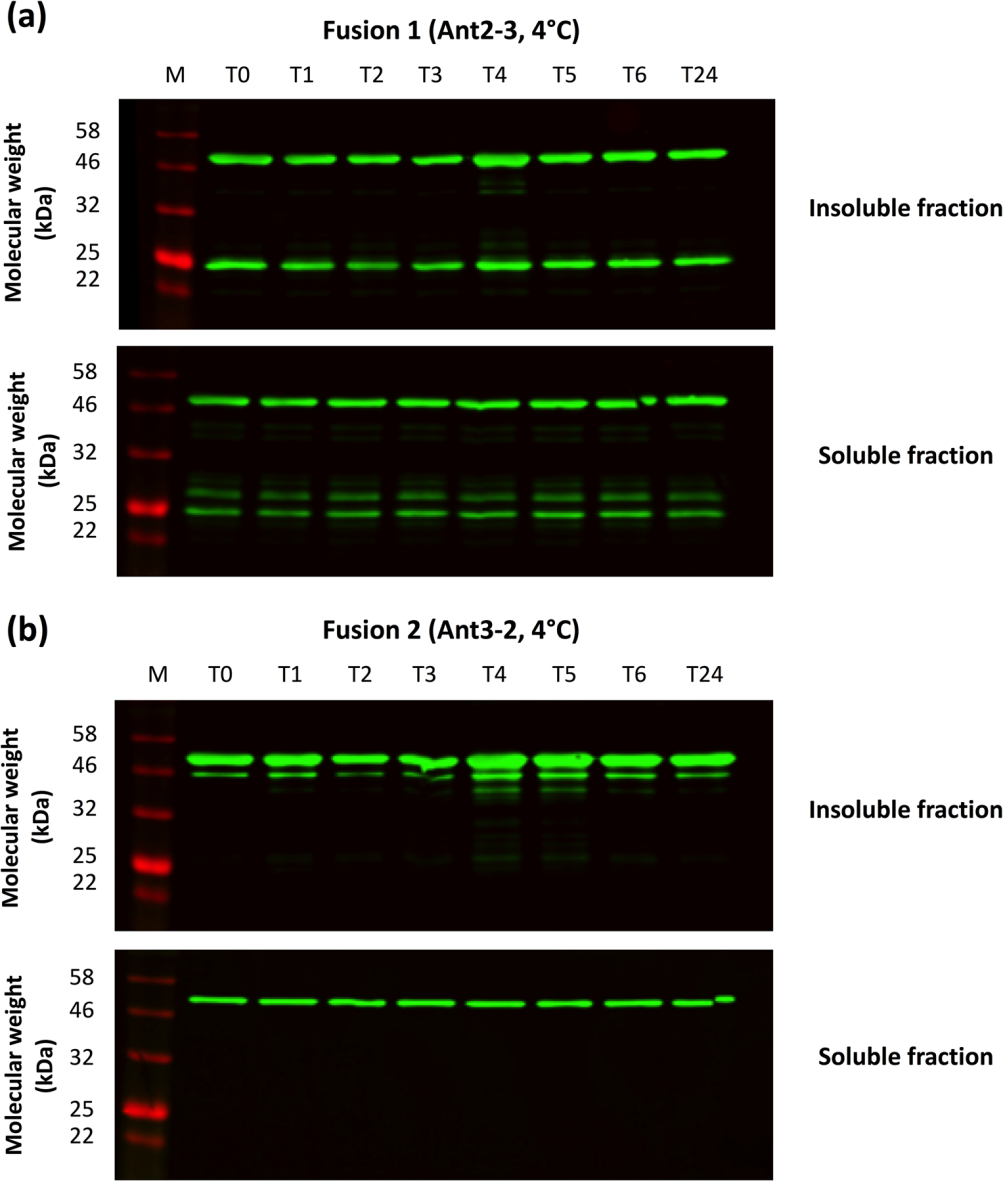
Assessment of protein stability of purified protein post-His tag purification. Fusion proteins were purified using the 6×His-tag via IMAC from the soluble and insoluble fraction. For both Ant2-3 (**a**) and Ant3-2 (**b**), the first elution sample (E1) from the purification of the insoluble and soluble fraction was sampled straight after elution (T0), then incubated at 4°C and sampled every hour over a 6-h time period (T1-T6) and after 24 h (T24). Samples were subsequently analysed via western blot using a using a 6×His tag primary antibody for detection

As improper processing of Ant2-3 was observed, further work was carried out to ascertain if cleavage of Ant2-3 was occurring post-lysis. Ant3-2 was also studied as a comparison. Both fusions were purified from the soluble and insoluble fraction, and purified samples were then incubated and sampled at different time points at 4°C for 24 h (Fig. 4) and 37°C for 2 h (data not shown) and analysed by western blotting.

Under both temperature conditions, no increase in the amount of degradation product was observed post-purification in the soluble and insoluble fraction for Ant2-3 (Fig. 4a). Further no proteolytic products of the same size were observed for Ant3-2 (Fig. 4b). The data suggested that the improper processing of Ant2-3 occurred during intracellular expression rather than post-lysis.

### Strategies to improve protein production and solubility

As a large proportion of all four antigens localised to the insoluble fraction and in the case of the fusions largely remained insoluble after re-folding, strategies were employed to improve protein production and solubility and prevent improper processing of fusion 1 (Ant2-3).

As mentioned earlier, different *E. coli* host strains were tested to assess their effect on the production and solubility of these target antigens. For example, the JM109-pGJKE8 (DE3) strain allowed the co-expression of chaperones alongside the target antigens with the aim to aid protein folding during expression. The data showed no improvement in the protein production and solubility. Further, proteolytic cleavage of Ant2-3 was observed in all strains (data not shown).

Alongside the chaperone strain studies, a heat-shock inducer benzyl alcohol (BA) was added to BL21-CodonPlus (DE3) cultures to improve recombinant protein yields and solubility and prevent aggregation. Previously, De Marco et al (De Marco et al. 2005) reported that the use of BA in cultures increased yields of recombinant targets comparable to the use of chaperone co-expression. Cultures transformed with Ant2, Ant3, Ant2-3 and Ant3-2 were treated with BA, 30 min prior to IPTG induction (18°C for 20 h). Post-induction cultures were harvested and protein production and solubility assessed for all antigens with and without BA addition (Figure S4). No significant difference was observed for cultures with BA compared to untreated cultures.

**Fig. 5:**
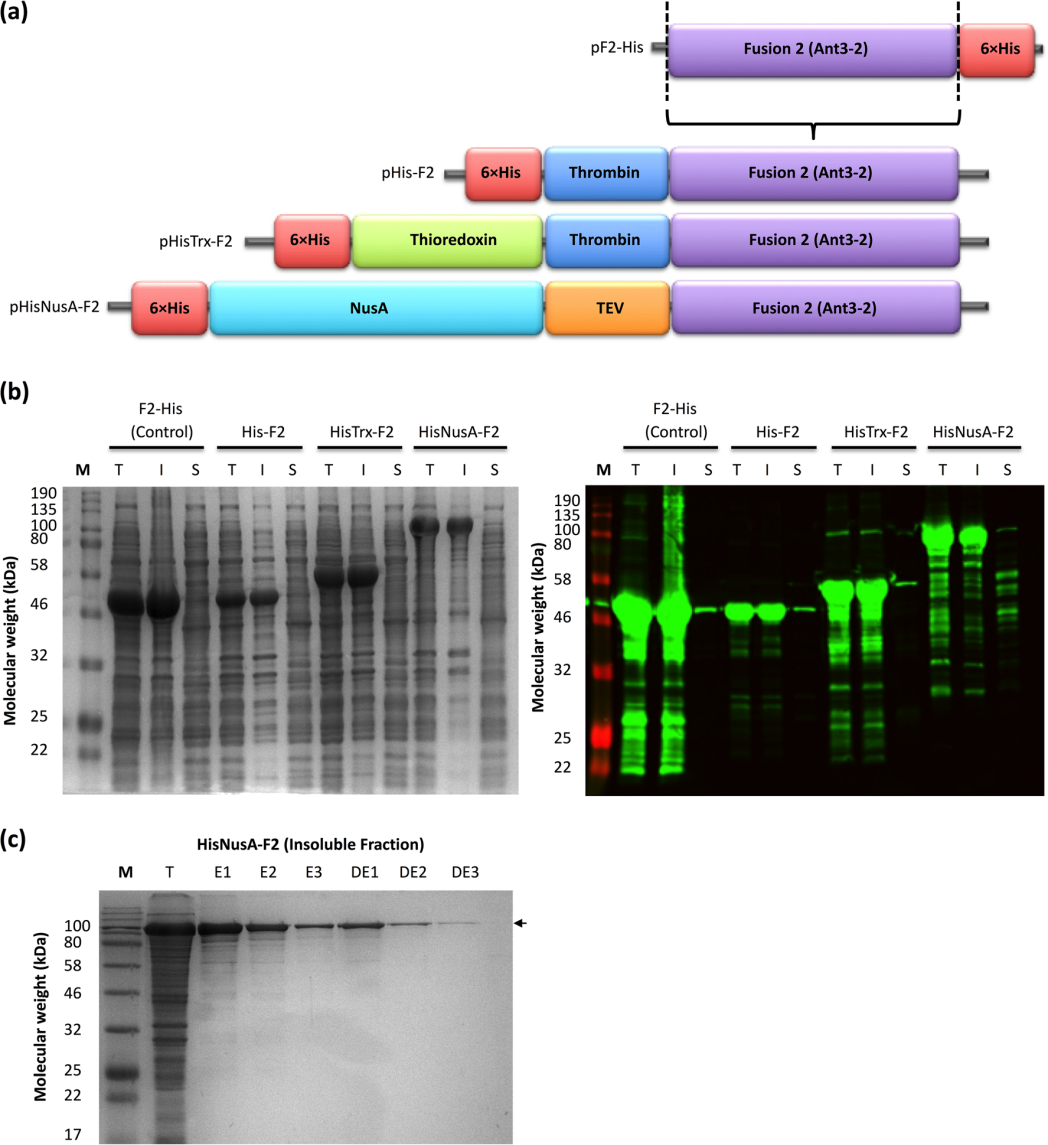
Addition of N-terminal solubility tags to improve protein solubility. The schematic diagram (**a**) summarises the three different constructs generated for fusion 2 (Ant3-2) with different cleavable N-terminal solubility tags. The non-modified Ant3-2 (control) and different N-terminally tagged Ant3-2 constructs were expressed in BL21-CodonPlus (DE3) *E. coli* strain and the protein production and solubility assessed via SDS-PAGE and western blot (**b**). pHisNusA-tagged Ant3-2 was also re-folded and purified via the 6×His-tag from the insoluble fraction (**c**)

An alternate strategy tested was the use of solubility tags. Initially, Ant3-2 (fusion 2) was used as a model, the idea was to test the effect on protein solubility and use the data to aid cloning of the appropriate/favourable tag with the other target antigens. The Ant3-2 gene sequence was cloned into pET16 vectors with different cleavable N-terminal solubility tags NusA and Trx (Fig. 5a). In addition, a N-terminal 6×His tag was present for detection. A N-terminally His-tagged Ant3-2 construct (pHis-F2) was generated as a control to account for the change in position of the His-tag from the original pET21d (pF2-His, C-terminal His-tag). Bacterial expression of the Ant3-2 constructs showed that the addition of the solubility tags did not increase the solubility of the antigens. Addition of the N-terminal His-tag resulted in a lower amount of total protein compared to the un-modified C-terminally His-tagged Ant3-2 (Fig. 5b).

The expression of NusA alone was used as a positive control (Figure S5a). NusA alone was mainly detected in the soluble fraction, whereas pNusA-Ant3-2 was insoluble. Though addition of the NusA did not increase solubility of Ant3-2 during bacterial expression, it did increase the amount of soluble protein after re-folding and His-tag purification (Fig. 5c). The N-terminal NusA tag was also added to Ant2, Ant3 and Ant2-3 to see if the lack of effect on protein solubility was specific to Ant3-2 or common across all four antigens. Addition of the N-terminal NusA tag did not increase solubility of the other antigens; rather, in all cases, a decrease in the amount of total protein was observed (Figure S5b).

As the strategies employed at the plasmid design and bacterial expression stage did not increase protein production and/or solubility, further work involved optimising the current methodology to keep purified material soluble. For purified Ant2 and Ant3 samples, post-purification from the insoluble fraction, protein precipitation was observed above a certain concentration range (1.5–2 mg/ml) either as proteins were eluted from the column or during storage at 4°C after 24–48 h, leading to significant loss in protein yields. To prevent such loss in protein yields, the solubility of purified material was assessed in different buffers for Ant2 and Ant3.

**Fig. 6:**
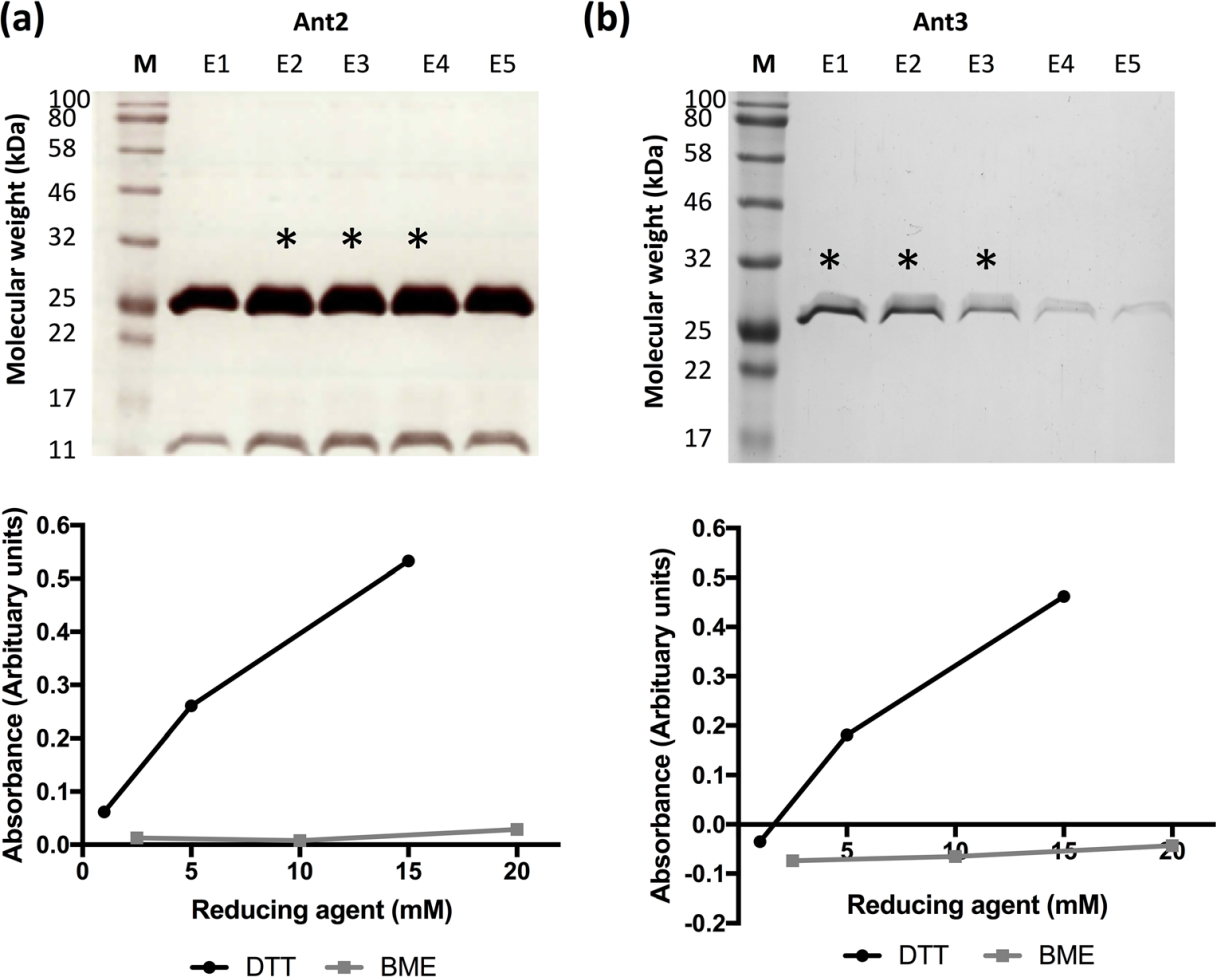
Protein solubilisation screen of the single antigens (Ant2 and Ant3). Ant2 (**a**) and Ant3 (**b**) were expressed in the BL21-CodonPlus (DE3) *E. coli* strain (1l total volume). Proteins were re-solubilised in urea buffer from the insoluble fraction and purified via the His-tag. Proteins were eluted with imidazole in 5 × 1 ml stages (E1-E5). The protein concentration was determined via the Bio-Rad DC protein assay kit (Table SI), and the top three concentrated samples (*) were pooled for the OptiSol™ protein solubility screen. Fifteen nicrolitres of protein were mixed with 150 μl of reagent per well and incubated at 37°C for 24 h (stressed conditions). Following incubation the soluble protein was collected by centrifugation. Absorbance readings (at 280 nm) were taken of the plate with reagent alone (blank) and after the collection of soluble protein following incubation under stressed conditions. The data was analysed and plotted for each condition using Protein Dashboard™. Data from the addition of reducing agents dithiothreitol (DTT) and β-mercaptoethanol (BME) is plotted for both Ant2 and Ant3 (**a**–**b**)

Ant2 and Ant3 were expressed in a total volume of 1 l and proteins re-folded and purified from the insoluble fraction (Fig. 6). The three eluate fractions with the highest protein concentration (determined by protein assay, Table SI) were pooled and analysed using the OptiSol™ protein solubility screening kit to identify conditions that could stabilise protein at higher concentrations after purification. Purified protein was plated out onto the supplied reagent plate and incubated under ‘stressed’ conditions (37°C for 24 h) and the amount of soluble protein remaining was assessed after incubation. Overall, Ant3 showed poorer stability compared to Ant2, as Ant3 precipitated under almost all conditions (negative absorbance values). Across the different conditions tested, the addition of reducing agent, dithiothreitol (DTT), had a significant effect on protein solubility for both Ant2 (Fig. 6a) and Ant3 (Fig. 6b). An increase in concentration of DTT (1–15 mM) resulted in a proportional increase in the amount soluble protein recovered after incubation. The addition of another reducing agent β-mercaptoethanol had a negligible effect on protein solubility. The data suggests that the addition of DTT alone may act to decrease the formation of insoluble aggregates and increase solubility of these antigens. This hypothesis was supported tested further as addition of DTT to the initial purified Ant2 sample and incubation under ‘stressed’ conditions showed an increase in the amount of soluble protein recovered compared to Ant2 without DTT (data not shown).

In addition to the strategies employed to increase protein yields and solubility, other work in parallel was completed to understand whether the lack of protein solubility could be predicted/rationalised based on the sequence and structural properties of these antigens. Further we aimed to assess whether sequence/structural features could guide the design of recombinant antigens to prevent limitations and aid efficient recombinant production.

### Analysis of the protein secondary structure

**Fig. 7:**
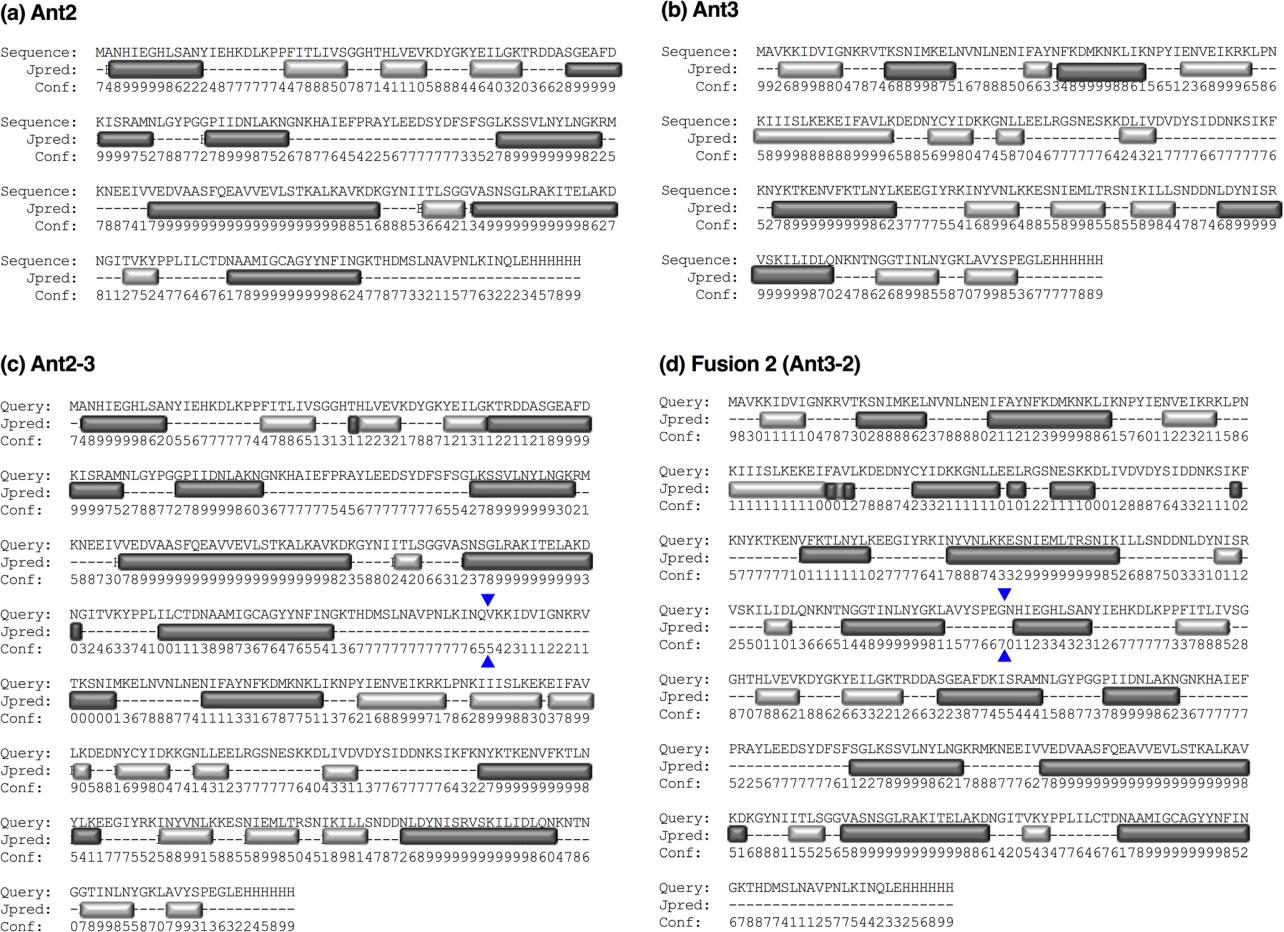
Amino acid based prediction of secondary structure. Using the amino acid sequence, a prediction of the secondary structure was generated using JPred 4 for Ant2 (**a**), Ant3 (**b**), fusion 1 (Ant2-3, **c**) and fusion 2 (Ant3-2, **d**). Predicted alpha helices (dark grey), beta strands (light grey) and no/other (-) types of structure are indicated. The boundaries between single antigens in fusion 1 and fusion 2 are indicated with blue arrowheads. An estimate of the confidence (conf) is shown, where a higher value is indicative of a high confidence estimate

Initial analysis of amino acid sequences centred on the prediction of secondary structural elements using the JPred 4 server (Fig. 7). Predictions of the single antigens (Fig. 7a–b) showed mostly ordered regions with differences in their secondary structure as expected. Analysis of the fusion proteins showed differences in the structure between Ant2-3 and Ant3-2 (Fig. 7c–d). Ant2-3 was predicted to contain a 30 amino acid un-structured region between the boundary of Ant2 and Ant3, whereas Ant3-2 was predicted to contain two alpha helices either side of the boundary between Ant3 and Ant2. The unstructured region in Ant2-3 could potentially result in improper processing and be the site of proteolytic cleavage as observed previously (Figs. 2, 3, and 4).

### Modelling and computational analysis of protein structures

Analysis of the amino acid sequence was performed to evaluate whether ‘difficulties’ in the expression of these proteins could be predicted. Amino acid sequence-based prediction using ProteinSol gave an average value below 1 for Ant2 (0.50) and Ant3 (0.81) and both fusions (0.56)—suggesting that all antigens were indeed soluble based on a database of all *E. coli* proteins expressed in a cell-free bacterial expression system. Therefore, sequence-based analysis of the antigens could not be used as a predictive tool of recombinant production of these antigens.

In parallel, structural models for Ant2 and Ant3 were generated based on their sequence homology to existing structures published in the protein data bank (PDB). Ant2 had 39% sequence identity to the published structure 4wq4A, whereas Ant3 had a lower homology at 15% with 2vh2A. It is important to note that these structures are predictions of these proteins and actual structures may vary. Models of the fusion proteins could not be generated, as both single antigens are not natural physiological partners.

**Fig. 8:**
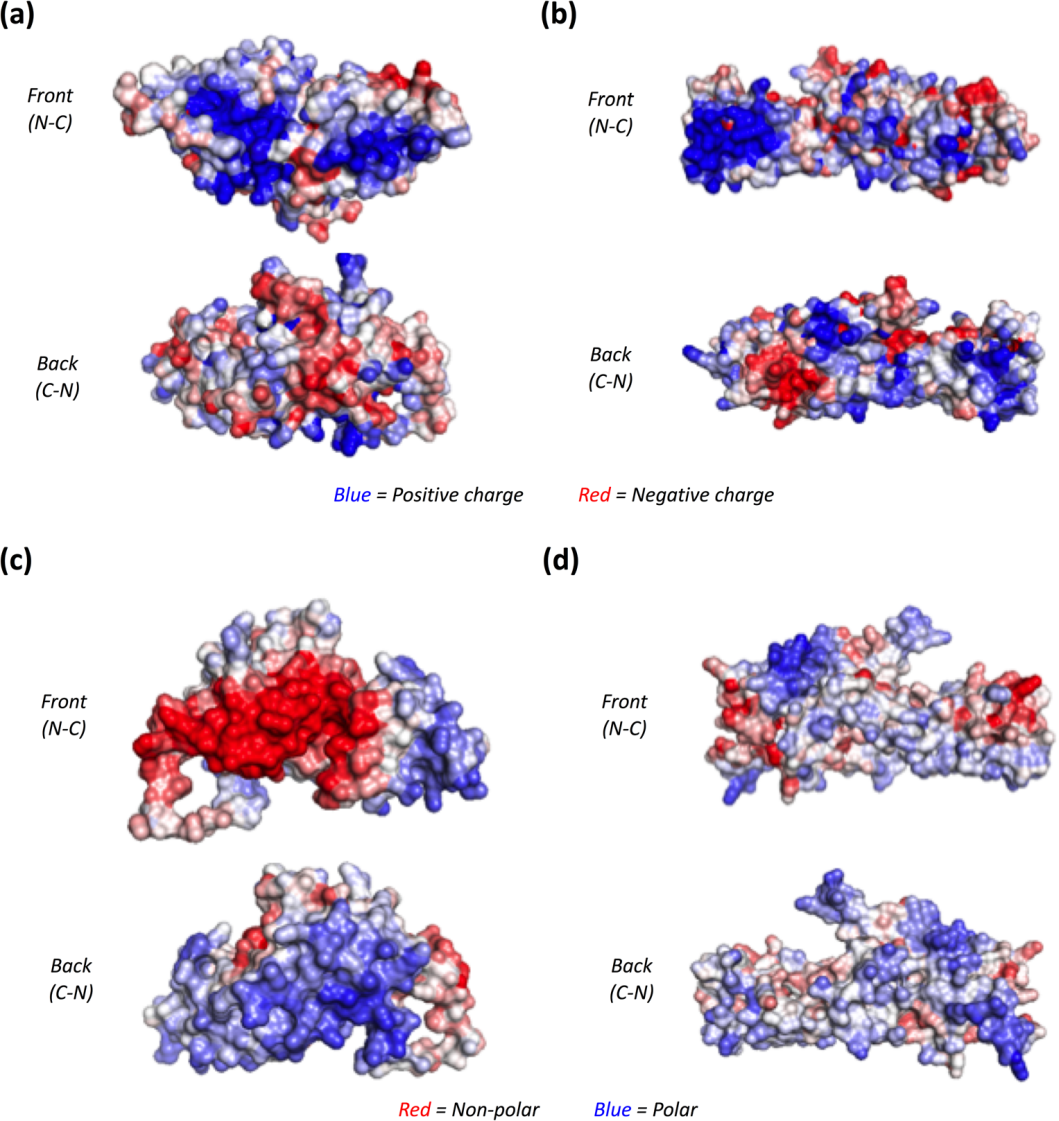
Computational analysis of the three-dimensional structure of single antigens. Structural models for Ant2 and Ant3 were generated using SWISS-MODEL where structures of homologs published in the protein data bank (PDB) for both Ant2 (PDB ID: 4wq4A) and Ant3 (PDB ID: 2vh2A) were used as a template. Structural models for Ant2 (**a**, **c**) and Ant3 (**b***,* **d**) were subsequently analysed by ProteinSol (Hebditch et al. 2017; Hebditch and Warwicker 2019). Separate predictions were generated of the surface electrostatic potential (negative- vs. positive-charge, **a**–**b**) and hydrophobicity (polar vs. non-polar, **c**–**d**). For all predictions, a front (N- to C-terminus from left to right, N-C) and back (C- to N-terminus from right to left, C-N) view is shown

Structural-based prediction showed that in terms of the electrostatic potential, Ant3 contained a larger positively charged patch (posQmax = 4125), which has shown to correlate to a poorer solubility (threshold = 2990) (Fig. 8b). Predictions for Ant2 (posQmax = 2781) were just within the threshold and considered soluble (Fig. 8a). Hydrophobicity analysis of both antigens showed that Ant2 has a notable non-polar patch (Fig. 8c), which could potentially have a negative impact on solubility. Ant3 showed no unfavourable features in terms of hydrophobicity (Fig. 8d).

## Discussion

In this case study, we have presented the expression and purification data for ‘difficult-to-express’ antigens from Absynth Biologic Ltd’s *C. difficile* programme. Two antigens, Ant2 and Ant3, were expressed as single antigens and as fusion (double-antigen) proteins to aid expression and decrease the cost-of-goods. Data shown in this study and observed by Absynth Biologics Ltd. showed that both single and fusion proteins formed insoluble aggregates when expressed in *E. coli*. Use of fusion proteins acted to improve the overall yield, but not the solubility of these recombinant targets.

**Fig. 9:**
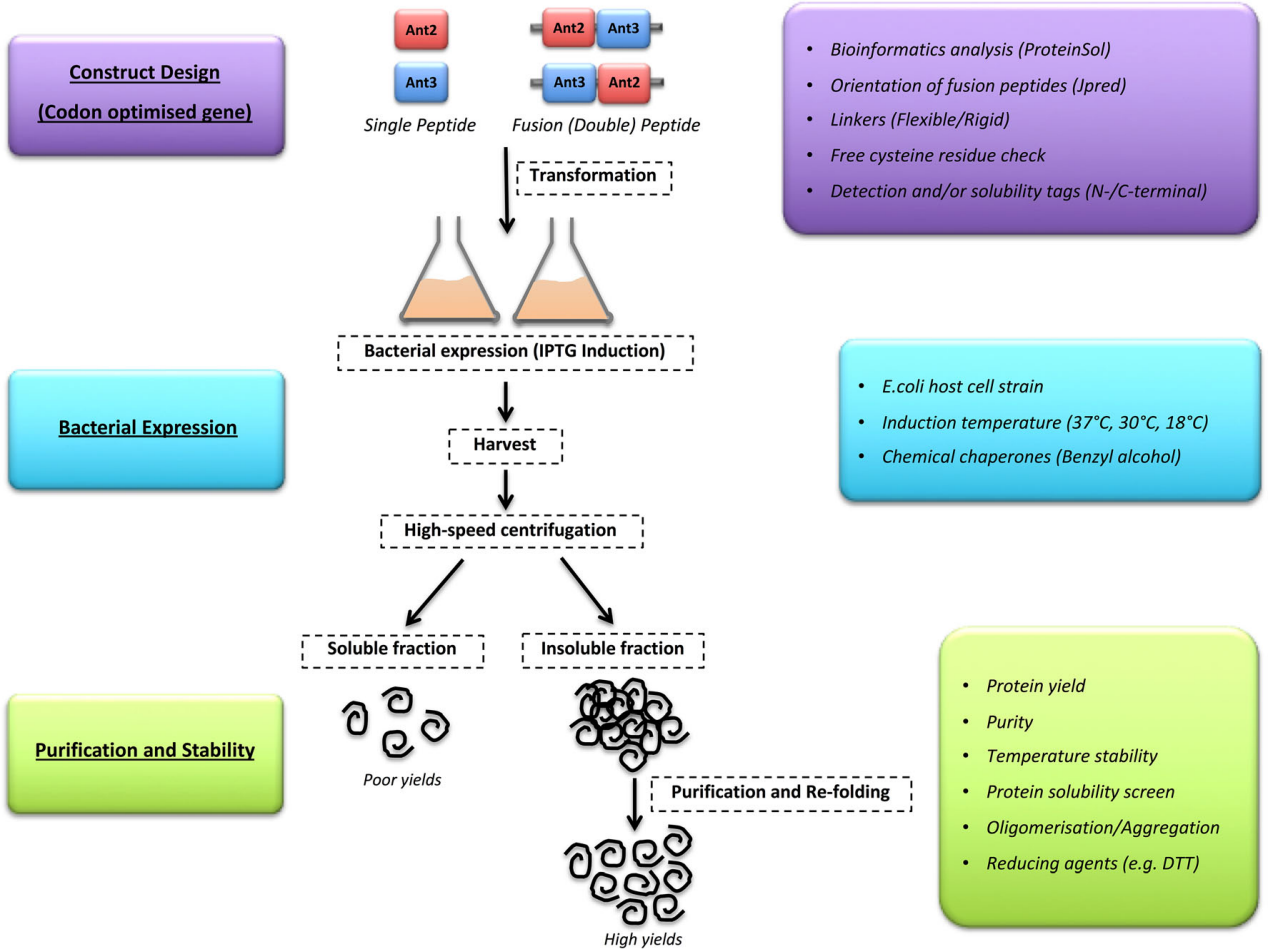
Flowchart and guide for the optimised expression and purification of recombinant protein production. The schematic summarises the methodology and observations from this study during the expression and purification of ‘difficult-to-express’ recombinant single and fusion antigens in *E. coli*. The workflow is separated into three stages construct design (purple), bacterial expression (blue) and purification and stability (green). For each stage, strategies and predictive tools to guide efficient recombinant production are listed (coloured boxes)

To overcome the limitations in the production of these ‘difficult’ antigens, widely used strategies were employed to improve bacterial recombinant protein production (Fig. 9). Further, the role of sequence and structural-based features on expression was analysed to help guide improved recombinant protein production (Fig. 9).

Initial efforts focused on the optimisation of bacterial culture conditions. A preliminary screen of different host *E. coli* strains showed no improvement in the solubility of these antigens. Further, no significant difference was observed between the bacterial cultures expressing all four antigens, suggesting that these antigens did not have a toxic effect and grew similarly between the single and fusion proteins. It was observed that a greater amount of soluble protein and less high and low molecular weight (predicted oligomers/degradation products) were detected at a low induction temperature (18°C), which was in agreement with other published studies (Chesshyre and Hipkiss 1989; Mizukami et al. 1986; Mujacic et al. 1999; Schein and Noteborn 1988; Vasina and Baneyx 1997). Purification studies showed a greater yield, and purity of proteins was achieved via purification and re-folding from the inclusion body–enriched insoluble fraction. The use of bacterial proteins as solubility tags has been a commonly used strategy to increase the production of recombinant targets. Commonly used tags include highly soluble Trx (Lavallie et al. 1993; LaVallie et al. 2000) and NusA (Davis et al. 1999; Li et al. 2013) bacterial proteins. Contrary to published reports, the solubility tags did not improve the overall solubility and processing of these antigens.

During the bacterial expression studies, it was observed that fusion 1 (Ant2-3) underwent proteolytic processing, whereas the alternative fusion Ant3-2 showed greater stability. Analysis of protein stability post-lysis suggested that the improper processing of Ant2-3 occurred during the bacterial expression stage rather than post-harvest of cultures. The use of an amino acid sequence-based secondary structure prediction tool, JPred 4, showed the presence of a long unstructured region between the protein boundaries in Ant2-3 and absent in fusion 2 (Ant3-2). Potentially the addition of a linker between Ant2 and Ant3 would prevent improper processing and aid in protein folding and hence solubility. Different flexible and rigid linkers have been utilised to construct fusion proteins and aid increased protein expression (Chen et al. 2013). Future design of fusion proteins or polypeptides (multiple antigens) could account for the presence of large unstructured regions, with the use of predictive tools prior to expression, to prevent downstream challenges in the expression of such recombinant antigens. Together, the mechanism by which these recombinant proteins formed insoluble aggregates and improper processing of fusion proteins occurred was not rescued by the optimisation of the bacterial expression conditions. As a result, increased efforts were applied to understand the implications of sequence and structural features on recombinant production of these ‘difficult-to-express’ antigens.

It was shown that the sequence and structure of these antigens are important considerations when designing antigens and fusions. Analysis of cysteine residues in both antigens showed the presence of two residues in Ant2 and a single unpaired cysteine in Ant3. As both antigens are fragments of a full-length protein, the corresponding cysteine pair for Ant3 was absent from this antigen. It is possible that these proteins may form di-sulphide bonds within and/or between separate protein molecules resulting in insolubility and the formation of the observed oligomers. Therefore, addition of DTT may act to prevent the formation of these di-sulphide bonds, therefore stabilising and increasing the solubility of these proteins. This hypothesis is supported by the increased solubility of Ant2 with DTT in the initial purified sample compared to Ant2 without DTT (data not shown). The identification and removal of redundant free cysteine residues could be included as an additional ‘checkpoint’ when designing recombinant antigens (Fig. 9). However, the physiological effect of removing such amino acid residues from antigens would need to be explored.

The use of a predictive solubility tool, ProteinSol, identified antigen-specific non-polar regions (Ant2) or positively charged (Ant3) on the surface of protein structures. These features alongside free cysteine residues may act alone and in combination with one another to negatively impact solubility. The structural-based prediction is based on a sequence homology-based model, and the native structure may vary, especially considering the antigens used in this study were taken from their full-length protein counterparts. Further, fusion proteins could not be modelled. Under physiological conditions, the charged/non-polar patches on these antigens may drive protein-proteins interactions, for example, with chaperones or specific interacting partners. Alternatively, these areas may drive oligomerisation with other interacting partners but under conditions of recombinant production of these antigens, these areas drive oligomerisation with one another resulting in insolubility. Potentially the re-design of peptide sequence to remove unfavourable attributes or mimicking physiological interacting partners in recombinant setting may drive increased solubility of these antigens. Further, integrating sequence analysis of potential sites important for activity and immunogenicity (T-cell and B-cell epitopes) would allow the design of optimised antigen sequences. For antigens that are ‘difficult-to-express’ despite these optimisation stages, the workflow could move to alternative strategies such as those described in this study (Fig. 9) to improve recombinant protein production.

In summary, we have presented the strategies and predictive tools employed to increase the production of ‘difficult-to-express’ antigens (Fig. 9). Whilst aware that the aims were not achieved due to commercial sensitivities, such studies are important in guiding others towards a critical understanding of potential limitations of specific approaches. We have highlighted the importance of screening antigen sequences and using predictive computational tools (JPred and ProteinSol), prior to expression studies, to guide the design of recombinant antigens. Such measures could potentially prevent limitations in their production and increase solubility and yields of ‘difficult’ recombinant targets.

## Supplementary information

ESM 1 (PDF 403 kb)
