# Supplementary material for: Predictive approaches to guide the expression of recombinant vaccine targets in Escherichia coli: a case study presentation utilising Absynth Biologics Ltd. proprietary Clostridium difficile vaccine antigens
Source: Appl Microbiol Biotechnol. 2021 Jun 28;105(13):5657–74. doi: 10.1007/s00253-021-11405-9 (PMC8285303; doi:10.1007/s00253-021-11405-9)
Supplement: Supplementary file 1 — (PDF 403 kb) [file 253_2021_11405_MOESM1_ESM.pdf]

Applied Microbiology and Biotechnology

Supplementary Material

Predictive approaches to guide the expression of recombinant vaccine targets in *Escherichia coli*: A case study presentation utilising Absynth Biologics Ltd proprietary *Clostridium difficile* vaccine antigens

Hirra Hussain[1], Edward A McKenzie[1], Andrew M Robinson[2†], Neill A Gingles[2††], Fiona Marston[2‡], Jim Warwicker[1] and Alan J Dickson[1*]

[1] Manchester Institute of Biotechnology, University of Manchester, M1 7DN, United Kingdom

[2] Absynth Biologics Ltd, BioHub, Alderley Park, Cheshire, SK10 4TG, United Kingdom

Now at:

† Evotec Limited, Biohub, Alderley Park, Cheshire, England, SK10 4TG, United Kingdom

†† metaLinear Limited, Biohub, Alderley Park, Cheshire, England, SK10 4TG, United Kingdom

‡ Liverpool School of Tropical Medicine, Liverpool, L3 5QA, United Kingdom

* Corresponding author: Professor Alan J Dickson,
Manchester Institute of Biotechnology
The University of Manchester
131 Princess Street
Manchester M1 7DN
United Kingdom
Email: alan.dickson@manchester.ac.uk

Ant2

MANHIEGHLSANYIEHKDLKPPFITLIVSGGHTHLVEVKDYGKYEILGKTRDDASGEAFDKISRAMNL
GYPGGPIIDNLAKNGNKHAIEFPRAYLEEDSYDFSFSGLKSSVLNYLNGKRMKNEEIVVEDVAASFQE
AVVEVLSTKALKAVKDKGYNIITLSGGVASNSGLRAKITELAKDNGITVKYPPLILCTDNAAMIGCAG
YYNFINGKTHDMSLNAVPNLKINQLEHHHHHH

Ant3

MAVKKIDVIGNKRVTKSNIMKELNVNLNENIFAYNFKDMKNKLIKNPYIENVEIKRKLPNKIIISLKE
KEIFAVLKDEDNYCYIDKKGNLLEELRGSNESKKDLIVDVDYSIDDNKSIKFKNYKTKENVFKTLNYL
KEEGIYRKINYVNLKKESNIEMLTRSNIKILLSNDDNLDYNISRVSKILIDLQNKNTNGGTINLNYGK
LAVYSPEGLEHHHHHH

Fusion 1 (Ant2-3)

MANHIEGHLSANYIEHKDLKPPFITLIVSGGHTHLVEVKDYGKYEILGKTRDDASGEAFDKISRAMNL
GYPGGPIIDNLAKNGNKHAIEFPRAYLEEDSYDFSFSGLKSSVLNYLNGKRMKNEEIVVEDVAASFQE
AVVEVLSTKALKAVKDKGYNIITLSGGVASNSGLRAKITELAKDNGITVKYPPLILCTDNAAMIGCAG
YYNFINGKTHDMSLNAVPNLKINQVKKIDVIGNKRVTKSNIMKELNVNLNENIFAYNFKDMKNKLIKN
PYIENVEIKRKLPNKIIISLKEKEIFAVLKDEDNYCYIDKKGNLLEELRGSNESKKDLIVDVDYSIDD
NKSIKFKNYKTKENVFKTLNYLKEEGIYRKINYVNLKKESNIEMLTRSNIKILLSNDDNLDYNISRVS
KILIDLQNKNTNGGTINLNYGKLAVYSPEGLEHHHHHH

Fusion 2 (Ant3-2)

MAVKKIDVIGNKRVTKSNIMKELNVNLNENIFAYNFKDMKNKLIKNPYIENVEIKRKLPNKIIISLKE
KEIFAVLKDEDNYCYIDKKGNLLEELRGSNESKKDLIVDVDYSIDDNKSIKFKNYKTKENVFKTLNYL
KEEGIYRKINYVNLKKESNIEMLTRSNIKILLSNDDNLDYNISRVSKILIDLQNKNTNGGTINLNYGK
LAVYSPEGNHIEGHLSANYIEHKDLKPPFITLIVSGGHTHLVEVKDYGKYEILGKTRDDASGEAFDKI
SRAMNLGYPGGPIIDNLAKNGNKHAIEFPRAYLEEDSYDFSFSGLKSSVLNYLNGKRMKNEEIVVEDV
AASFQEAVVEVLSTKALKAVKDKGYNIITLSGGVASNSGLRAKITELAKDNGITVKYPPLILCTDNAA
MIGCAGYYNFINGKTHDMSLNAVPNLKINQLEHHHHHH

Legend: Ant2 Ant3 Linker His-tag

Figure S1: Amino acid sequences for all antigens: The amino acid sequence for single antigens, Ant2 (blue, WP_021389366), Ant3 (black, WP_131025834.1), fusion 1 (Ant2-3) and fusion 2 (Ant3-2) are shown. The position of a linker (green) and C-terminal 6×His tag (underlined) is also indicated.

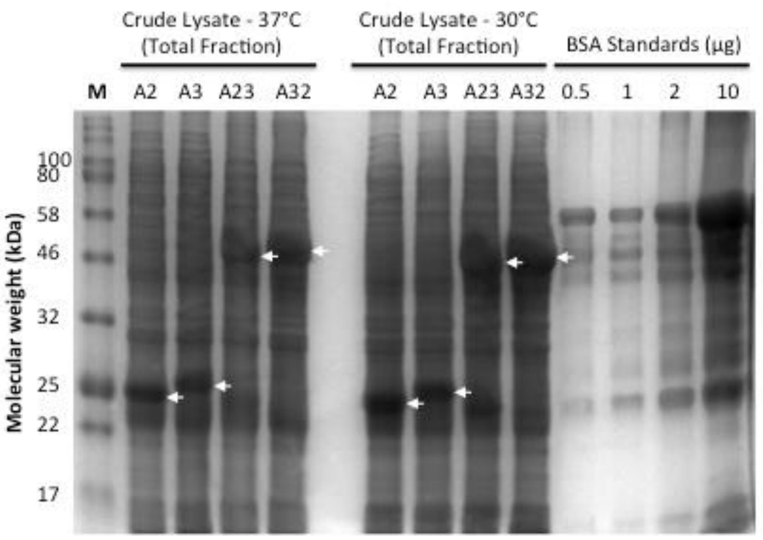

Figure S2: Analysis of protein production in bacterial cultures grown at higher temperatures (37°C and 30°C): Bacterial cultures for each antigen were harvested post-IPTG induction (20 h at 37°C or 30°C). The cell pellet was lysed via sonication and a sample of the crude lysate (total fraction) analysed by SDS-PAGE. Bovine serum albumin (BSA) was used as a protein standard. The band position for each protein is indicated with an arrow. Data is representative of at least four biological replicates.

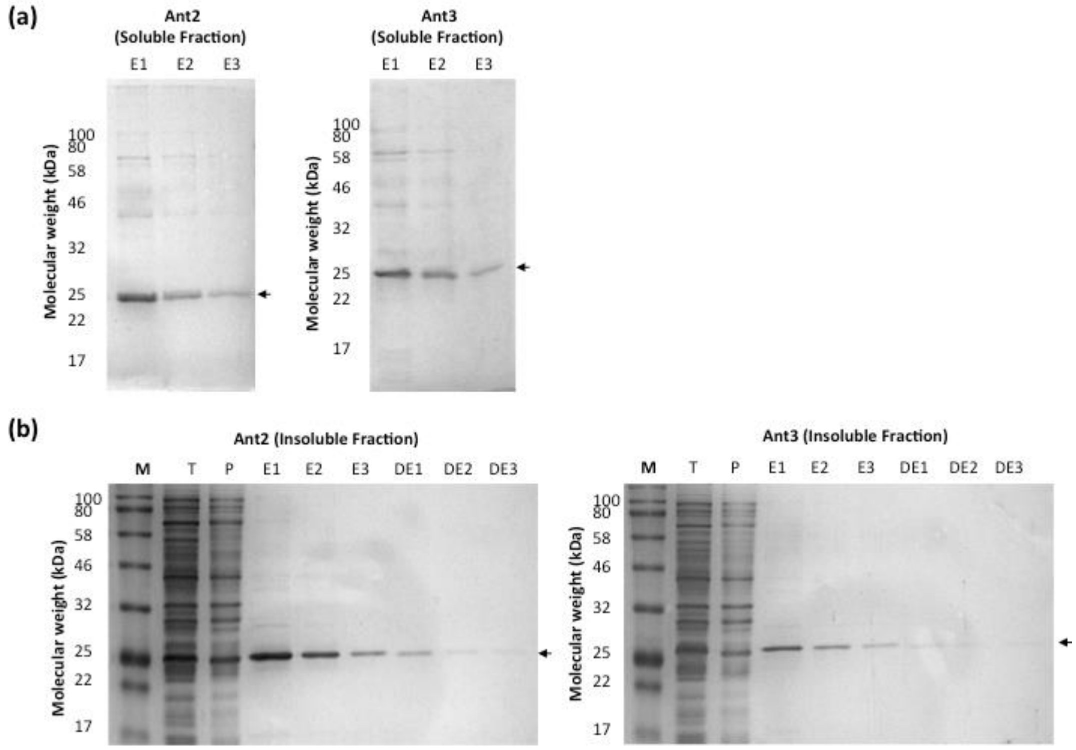

Figure S3: Purification of the single antigens from the soluble and insoluble fraction using immobilised metal affinity chromatography (IMAC). Ant2 and Ant3 were purified using the 6×His tag via IMAC from the soluble (**a**) and insoluble (**b**) fraction. For the insoluble fraction, proteins were re-solubilised in urea buffer prior to His-tag purification. Proteins were eluted with imidazole in 3 x 1 ml stages (E1-E3). For the insoluble fraction, after elution with imidazole (E1-E3), insoluble proteins (after the re-solubilisation stage) were eluted with in 3 x 1 ml stages with a denaturing elution buffer (DE1-DE3).

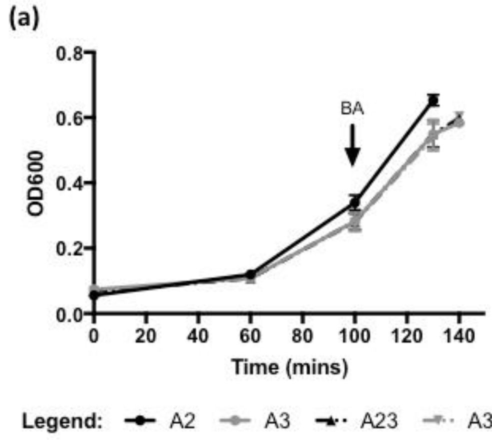

Target	Ant2	Ant3	Ant2-3	Ant3-2
Doubling time (mins)	30.92	35.44	34.62	34.46

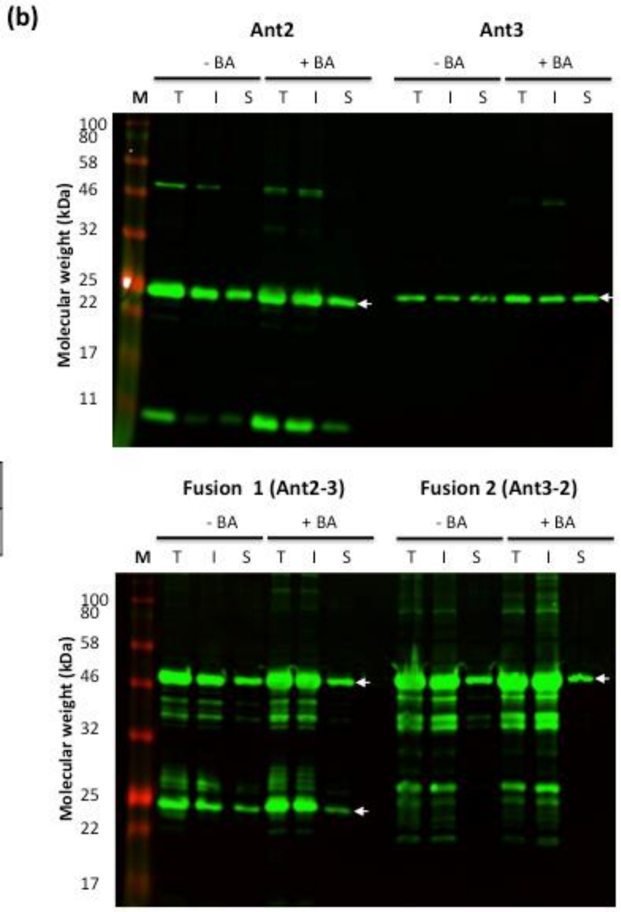

Figure S4: Addition of benzyl alcohol to bacterial cultures. This figure shows the OD600 plotted against the time (mins) for bacterial cultures transformed with Ant2, Ant3, fusion 1 (Ant2-3) and fusion 2 (Ant3-2) (**a**). Benzyl alcohol (BA, 10 mM final concentration) was added to cultures 30 min prior to IPTG induction. The table summarises the estimated doubling time (mins) of each culture analysed using the exponential growth equation in GraphPad Prism. Error bars shown are the mean value ± SEM of three biological replicates (n=3). Untreated cultures were used as a control (data not shown). Cultures were induced with IPTG and incubated for 20 h at 18°C. After incubation, cultures ± BA were harvested and the total fraction (T), insoluble fraction (I) and soluble fraction (S) isolated for western blot analysis (**b**). Data is representative of three biological replicates.

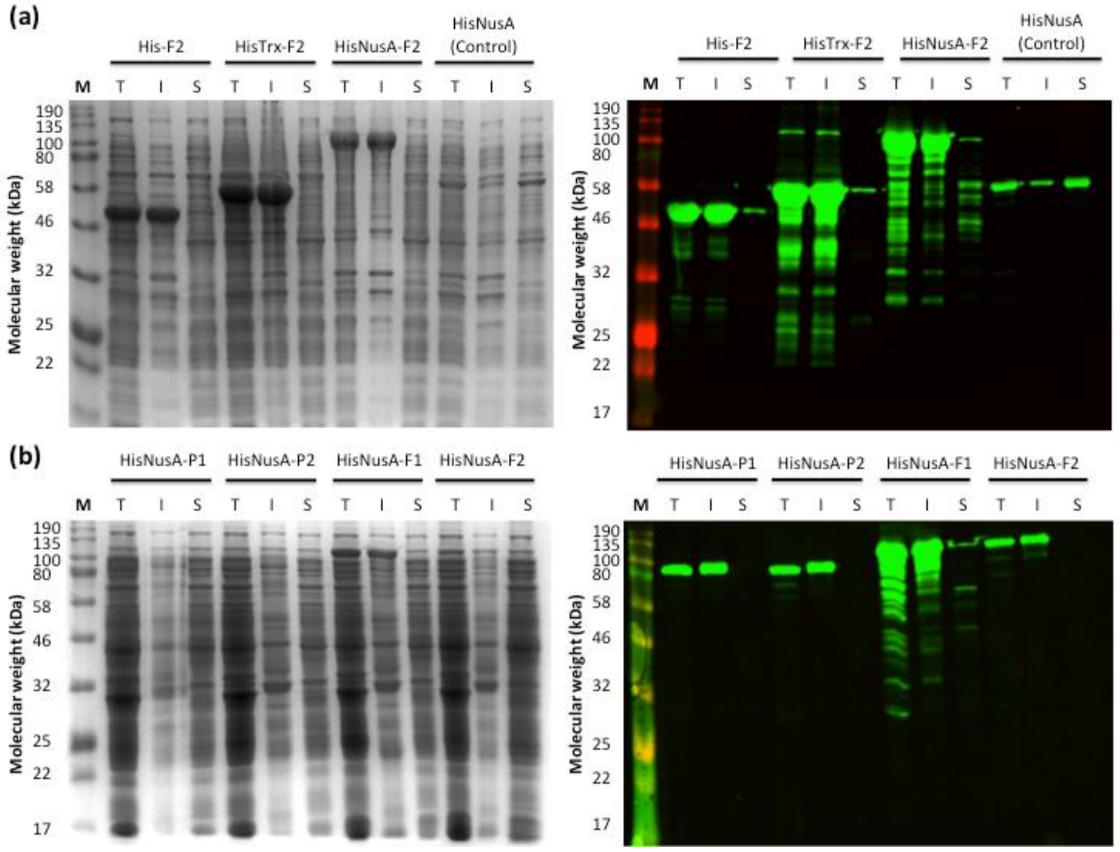

Figure S5: Repeat analysis of N-terminal solubility-tagged Fusion 2 (Ant3-2) constructs and addition of the N-terminal NusA solubility tag to the single antigens (Ant2 and Ant3) and fusion 1 (Ant2-3). This figure shows repeat SDS-PAGE and western blot analysis of the different N-terminally tagged Ant3-2 constructs expressed in the BL21-CodonPlus (DE3) *E. coli* strain with the expression of pHisNusA alone as a control (**a**). Ant2 (P1), Ant3 (P2) and Fusion 1 (F1, Ant2-3) were cloned with a N-terminal pHisNusA tag. The different N-terminally tagged constructs were expressed in BL21-CodonPlus (DE3) *E. coli* strain and the protein production and solubility assessed via SDS-PAGE and western blot (**b**).

Table SI: Protein concentration of purified protein samples.

This table summarises the estimated protein concentration for Ant2 and Ant3 after 6×His tag purification and re-folding from the insoluble fraction (1 L total volume bacterial culture).

**Sample**		**Protein concentration (mg/ml)**
**Ant2**	*E1*	1.52
	E2	1.80
	E3	1.99
	E4	1.94
	E5	1.71
**Ant3**	*E1*	1.09
	E2	1.16
	E3	1.20
	E4	1.13
	E5	1.12
